# PARADOXICAL FINDINGS AND OTHER SURPRISES IN PROVIDING STREET MEDICINE TO UNSHELTERED HOMELESS OLDER ADULTS

**DOI:** 10.1093/geroni/igad104.1005

**Published:** 2023-12-21

**Authors:** Alexis Coulourides Kogan, Anuva Mittal, Enya Lowe, Corinne Feldman

**Affiliations:** University of Southern California, Los Angeles, California, United States; Keck School of Medicine of USC, Los Angeles, California, United States; University of Southern California, Keck School of Medicine of USC, Los Angeles, California, United States; University of Southern California, Keck School of Medicine of USC, Los Angeles, California, United States

## Abstract

Older adults constitute the fastest-growing segment of the homeless population in the US. Previous research has found older homeless adults to have more chronic conditions, greater odds of physical disability, and experience accelerated aging compared to housed populations. Little is known about the role of street medicine in providing care and support to aged and aging patients experiencing unsheltered homelessness. The purpose of this study was to elicit the perspective of clinicians and unsheltered homeless patients on aging and managing serious illness. We conducted interviews with patients receiving street medicine and their clinicians. Interviews were guided by a semi-structured protocol, audio recorded, and transcribed verbatim. Data were analyzed following a thematic analysis approach. Eight clinicians and eight patients were interviewed. On average, clinicians were 41 years old, white (50%), and female (50%) with 1-16yrs street medicine experience. Patients were commonly male (63%), had 3+ chronic conditions (100%), and aged 56 years. Thematic analysis revealed four major paradoxical themes around: End-of-life, shelter, durable medical equipment, and community. The clinician and patient perspective on aging and managing serious illness while living outside offer insight into the significant and paradoxical challenges each face when balancing restrictions from the healthcare system, social services, and patient/self needs. Results highlight constraints placed on clinicians and patients by the rigid healthcare system and its incompatibility with the unique circumstances of unsheltered older adults. Findings from this study suggest actionable strategies that hold implications for policy and practice to better meet the needs of unsheltered homeless older adults.

